# Adipose Tissue Quantification Improves the Prognostic Value of GLIM Criteria in Advanced Gastric Cancer Patients

**DOI:** 10.3390/nu16050728

**Published:** 2024-03-02

**Authors:** Geum Jong Song, Hyein Ahn, Myoung Won Son, Jong Hyuk Yun, Moon-Soo Lee, Sang Mi Lee

**Affiliations:** 1 Department of Surgery, Soonchunhyang University Cheonan Hospital, 31 Suncheonhyang 6-gil, Dongnam-gu, Cheonan 31151, Republic of Korea; gjsong@schmc.ac.kr (G.J.S.); mwson@schmc.ac.kr (M.W.S.); 109206@schmc.ac.kr (J.H.Y.); 2 Department of Pathology, CHA Gangnam Medical Center, CHA University School of Medicine, Nonhyon-ro 569, Gangnam-gu, Seoul 06135, Republic of Korea; 3 Department of Nuclear Medicine, Soonchunhyang University Cheonan Hospital, 31 Suncheonhyang 6-gil, Dongnam-gu, Cheonan 31151, Republic of Korea

**Keywords:** gastric cancer, GLIM criteria, malnutrition, prognosis, visceral adipose tissue

## Abstract

The present study investigated whether the risk of recurrence after curative surgery could be further stratified by combining the Global Leadership Initiative on Malnutrition (GLIM) criteria and changes in subcutaneous (SAT) and visceral (VAT) adipose tissue mass after surgery in patients with advanced gastric cancer (AGC). This study retrospectively analyzed 302 patients with AGC who underwent curative surgery. Based on the GLIM criteria, patients were classified into malnourished and non-malnourished groups. The cross-sectional areas of SAT and VAT were measured from preoperative and 6-month post-operative computed tomography (CT) images. Multivariate survival analyses demonstrated that GLIM-defined malnutrition (*p* = 0.008) and loss of VAT after surgery (*p* = 0.008) were independent risk factors for recurrence-free survival (RFS). Evaluation of the prognostic value of combining the two independent predictors showed that malnourished patients with a marked loss of VAT had the worst 5-year RFS rate of 35.2% (*p* < 0.001). Preoperative GLIM-defined malnutrition and a loss of VAT during the first 6 months after surgery were independent predictors for RFS in patients with AGC. Changes in the VAT area after surgery could further enhance the prognostic value of the GLIM criteria for predicting the risk of gastric cancer recurrence.

## 1. Introduction

Despite a declining incidence rate in recent decades, gastric cancer remains a major cancer globally, ranking fifth in incidence and fourth in mortality [1]. Gastric cancer can be classified according to the depth of the cancer invasion, namely early gastric cancer (cancers with mucosal or submucosal layer invasion) and advanced gastric cancer (AGC; cancers with invasion beyond the proper muscle layer) [2]. For AGC patients with no distant metastasis, radical gastrectomy and regional lymph node dissection with/without adjuvant chemotherapy are recommended as the standard curative treatment [2]. However, even though patients receive curative surgical resection with optimal regional lymph node assessment, the prognosis of patients with AGC remains dismal, with a median overall survival of only 34 months [3]. Hence, prognostic factors that can predict the risk of cancer progression are needed, which can lead to an optimal treatment strategy selection [4].

Along with tumor-related prognostic factors such as tumor stage and histopathological classification, nutritional status at the time of cancer diagnosis is known to be significantly associated with clinical outcomes in patients with gastric cancer [5,6]. Reflecting the clinical significance of nutritional status, various diagnostic tools have been used to determine the nutritional status of patients; however, there is a fundamental lack of consensus on the diagnostic criteria for malnutrition that applies in clinical settings [6,7]. To overcome this problem, the Global Leadership Initiative on Malnutrition (GLIM) criteria, which comprises two diagnostic assessment criteria, phenotypic (weight, body mass index, and muscle mass) and etiologic (food intake and inflammation), have been proposed as a global consensus for diagnosing malnutrition in adults [7]. Based on the GLIM criteria, malnutrition is found to be quite common in patients with gastric cancer, showing incidence rates between 36.3 and 69.2% [6,8,9]. In addition, among patients with gastric cancer, those who were malnourished before treatment showed significantly worse prognoses than those with no malnutrition [6,9,10].

Recent studies of gastric cancer demonstrated that changes in body composition parameters after treatment could serve as a prognostic factor [5,11,12]. Due to nutritional malabsorption and gastrointestinal hormonal changes after gastric cancer surgery, most patients with gastric cancer experience post-operative weight loss, with the most prominent weight loss taking place in the first 6 months following the surgery [13,14]. For this reason, a significant portion of patients also experience a diminishment of the amount of subcutaneous (SAT) and visceral (VAT) adipose tissue after gastric cancer surgery [5]. In previous studies, loss of SAT and VAT after treatment was shown to be significantly associated with worse clinical outcomes in gastric cancer patients, suggesting that changes in nutritional status after treatment as well as initial nutritional status could be significant host factors for predicting prognosis [5,11]. Therefore, combining the initial nutritional status with changes in nutritional status after treatment could have further clinical significance in prognostic stratification.

In the present study, we measured the SAT and VAT mass from preoperative and 6-month post-operative computed tomography (CT) images in AGC patients treated with curative radical surgery and assessed the prognostic significance of the GLIM criteria and changes in SAT and VAT mass between preoperative and post-operative CT scans, as parameters reflecting the initial nutritional status and changes in nutritional health. We also evaluated whether changes in adipose tissue mass could further improve the predictive value of the GLIM criteria for recurrence-free survival (RFS).

## 2. Materials and Methods

### 2.1. Study Population

The electronic medical records of 449 patients with AGC who underwent surgery at Soonchunhyang University Cheonan Hospital between January 2011 and April 2021 were retrospectively reviewed. Among them, we enrolled patients who (1) showed no distant metastasis (stage M0) on staging imaging examinations, (2) received curative radical surgery, and (3) underwent an abdominopelvic CT scan both before surgery and 6 months after surgery. The patients who (1) had a previous history of another malignant disease or major abdominal surgery, (2) received palliative surgery or neoadjuvant treatment before surgery, (3) had no post-operative CT scan at 6 months after surgery, (4) had inadequate CT images for measuring skeletal muscle and adipose tissue areas, and (5) did not show for follow-up within 2 years after surgery without event were excluded from the study. Based on the inclusion and exclusion criteria, a total of 302 patients were finally enrolled in this study.

All enrolled patients underwent staging work-up examinations before curative surgery, including physical examinations, blood tests, gastroduodenoscopy, and abdominopelvic CT. Based on the height and weight measurements at the staging work-up, the body mass index (BMI) was calculated for each patient and categorized according to Asian standards: underweight < 20.0 kg/m^2^; normal 20.0–24.9 kg/m^2^; and obese ≥ 25.0 kg/m^2^ [5]. All patients received radical gastrectomy with D2 lymphadenectomy; pathological staging was determined according to the American Joint Committee on Cancer staging guidelines. Following the surgery, adjuvant chemotherapy was recommended based on the pathological staging and the patient’s clinical condition. Clinical follow-ups were regularly conducted at intervals of 6–8 months for the first 3 years and 10–12 months thereafter with blood tests, gastroduodenoscopy, and contrast-enhanced abdominopelvic CT.

### 2.2. Measurement of Skeletal Muscle and Adipose Tissue

**Figure 1:**
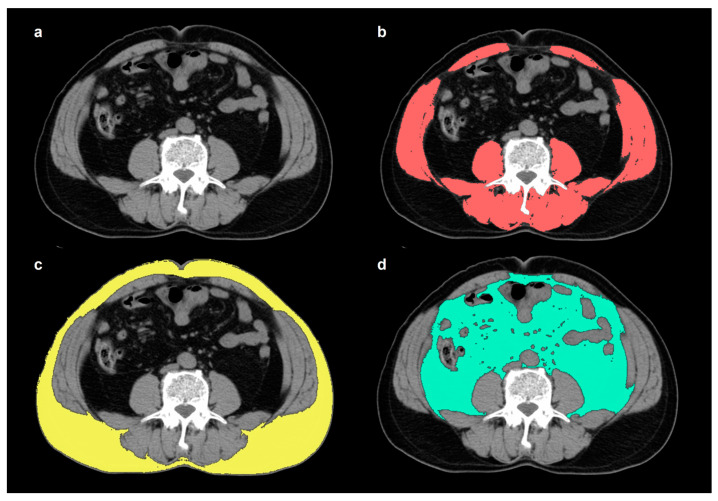
An example of the method used to calculate total skeletal muscle, SAT, and VAT areas from non-contrast-enhanced abdominopelvic CT scan images. On a transaxial CT image at the level of the L3 spine (**a**), the total skeletal muscle (**b**) area was automatically delineated using a CT-attenuation range from −29 to 150 HU (scarlet color in (**b**)). A CT-attenuation threshold of −190 to −30 HU was used for both SAT and VAT. SAT (**c**) was defined as extra-peritoneal adipose tissue between the skin and muscle (yellow color in (**c**)), while VAT (**d**) was defined as intra-abdominal adipose tissue (light green color in (**d**)).

From preoperative and 6-month post-operative abdominopelvic CT scans (GoldSeal CT750, GE Healthcare, Chicago, IL, USA), non-contrast-enhanced images were retrospectively reviewed to measure body compositions. The median interval between preoperative CT and surgery and between surgery and 6-month follow-up CT were 18 days (range, 2–55 days) and 6.7 months (range, 4.9–10.7 months), respectively. Using a United States Food and Drug Administration-approved imaging viewer (OsiriX 10.0.3 software, Pixmeo, Geneva, Switzerland), total skeletal muscle, SAT, and VAT areas were measured on a single transaxial CT image at the level of the L3 spine (Figure 1). The total skeletal muscle area (cm^2^) was comprised of the psoas, lumbar erector spinae, quadratus lumborum, transversus abdominis, internal and external oblique, and rectus abdominis muscles, which were delineated with a CT-attenuation threshold of −29 to 150 Hounsfield units (HU) [9,15]. Using a CT-attenuation threshold of −190 to −30 HU, the SAT and VAT areas (cm^2^) were measured [16,17]. All values of total skeletal muscle, SAT, and VAT areas were normalized for height squares (m^2^), which were defined as the skeletal muscle index, SAT index, and VAT index, respectively (cm^2^/m^2^). For the SAT and VAT indices, the percent change between the initial preoperative and post-operative 6-month follow-up CT scans was calculated as follows; (Δindex) = ([index on 6 months follow-up CT scan] − [index on initial CT scan])/(index on initial CT scan) × 100 (%). A total of six adipose tissue index parameters were analyzed in this study: initial SAT index, initial VAT index, follow-up SAT index, follow-up VAT index, ΔSAT index, and ΔVAT index.

### 2.3. GLIM Criteria

The nutritional status before surgery was assessed for the enrolled patients according to the GLIM criteria. Patients were assigned to the malnourished group when they fulfilled at least one phenotypic criterion and one etiologic criterion, and the remainder were assigned to the non-malnourished group [7]. The phenotypic criteria were comprised of weight loss (>5% within the past 6 months or >10% beyond 6 months), low body index (<18.5 kg/m^2^ if <70 years or <20 kg/m^2^ if >70 years), and reduced muscle mass (skeletal muscle index of <52.4 cm^2^/m^2^ for men and <38.5 cm^2^/m^2^ for women) [7,9,15]. The etiologic criteria included reduced food intake (≤50% of energy requirements >1 week, any reduction for >2 weeks, or any gastro-intestinal condition that adversely impacts food absorption) and inflammation (acute disease/injury or chronic disease-related condition such as malignant disease) [7].

### 2.4. Statistical Analysis

**Figure 2:**
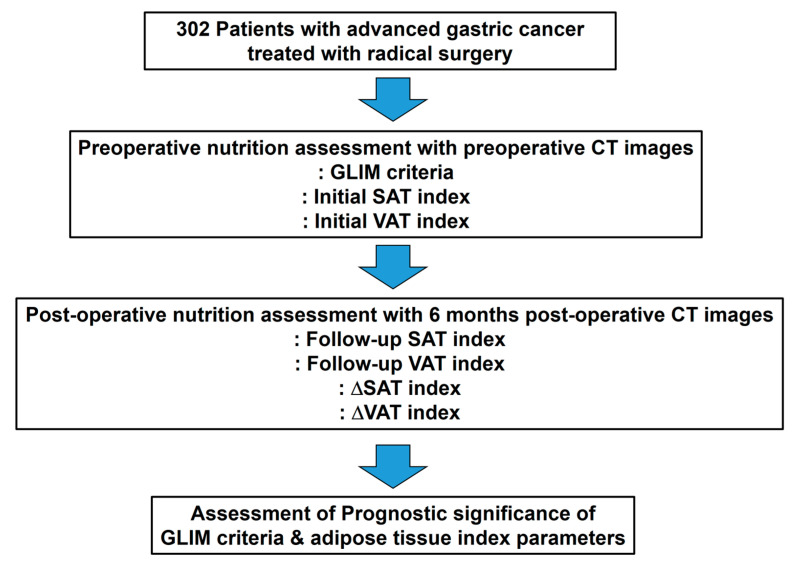
Schematic overview of the overall workflow in the study.

A schematic presentation of the workflow in the present study is depicted in Figure 2. Data were checked for normality using the Shapiro–Wilk test. Differences in variables between patient subgroups were compared using the Mann–Whitney U test, Chi-square test, Fisher’s exact test, or Kruskal–Wallis test with post-hoc analysis using Dunne’s test. The primary endpoint of this study was recurrence-free survival (RFS), which was defined as the time interval from the day of surgery to the day of gastric cancer recurrence detection or death. Patients with no events were censored on the day of the last clinical follow-up. Univariate and multivariate Cox proportional hazard regression analyses were conducted to investigate the prognostic significance of the variables for predicting RFS. Receiver operating characteristic (ROC) curve analysis was used to determine the optimal cut-off values of the continuous variables for survival analysis. For the multivariate survival analysis, only statistically significant parameters from the univariate analysis were selected. The cumulative RFS curves were estimated using the Kaplan–Meier method and analyzed by a log-rank test. All statistical analyses were performed using the MedCalc Statistical Software version 22.007 (MedCalc Software Ltd., Ostend, Belgium). The level of statistical significance was set at *p* < 0.05.

## 3. Results

### 3.1. Clinicopathological Characteristics of the Patients

The baseline clinicopathological characteristics of the 302 enrolled patients with AGC are listed in Table 1. In the overall population, lymph node metastasis was found in 175 patients (57.9%), while 117 patients (38.7%) had a stage III tumor. According to the GLIM criteria, 70 patients (23.2%) were diagnosed with malnutrition and the remaining 232 patients (76.8%) were assigned to the non-malnourished group. After surgery, 263 patients (87.1%) received adjuvant chemotherapy. On the 6-month follow-up CT images, 233 patients (77.2%) and 270 patients (89.4%) showed decreased SAT and VAT index values, respectively, when compared to preoperative CT images. The median values of the ΔSAT and ΔVAT indices were −26.3% and −52.0%, respectively.

The median post-operative follow-up duration was 56.8 months (range, 6.9–144.3 months). During the follow-up, 75 patients (24.8%) had events. The 5-year RFS rate of the enrolled patients was 75.6% (95% CI, 70.5–80.7%). A comparative analysis between the patients with and without events showed that patients with events had more advanced tumor stages and had a significantly higher proportion of malnourished patients (*p* < 0.05; Table 1). Furthermore, patients with events showed significantly lower values of follow-up SAT index, follow-up VAT index, ΔSAT index, and ΔVAT index (*p* < 0.05; Table 1).

In comparisons of adipose tissue index parameters according to the ages of the patients, significant differences in the initial SAT index, follow-up VAT index, ΔSAT index, and ΔVAT index were shown (Table S1). In the post-hoc analysis, patients with <50 years had significantly higher values of initial SAT index than those with 50–70 years (*p* < 0.05). In contrast, patients with <50 years showed significantly lower values of the follow-up VAT index, ΔSAT index, and ΔVAT index than those with >70 years (*p* < 0.05).

### 3.2. Survival Analysis for RFS

The prognostic significance of the GLIM criteria and adipose tissue parameters on CT images for predicting RFS was assessed using univariate and multivariate Cox regression analysis. All continuous variables included in the survival analysis other than age were dichotomized by the specific cut-off values determined by the ROC curve analysis: 43.6 cm^2^/m^2^ for the initial SAT index, 39.5 cm^2^/m^2^ for the initial VAT index, 29.5 cm^2^/m^2^ for the follow-up SAT index, 11.1 cm^2^/m^2^ for the follow-up VAT index, −41.6% for the ΔSAT index, and −62.0% for the ΔVAT index. Regarding age, patients were classified into three groups: (1) <50 years; (2) 50–70 years; and (3) >70 years.

**Figure 3:**
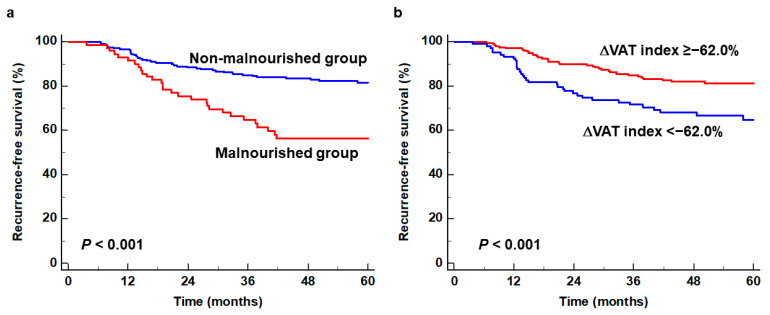
Cumulative recurrence-free survival curves based on the GLIM criteria (**a**) and the ΔVAT index (**b**).

In the univariate survival analysis, GLIM-defined malnutrition, the initial VAT index, the follow-up SAT index, the follow-up VAT index, the ΔSAT index, and the ΔVAT index were significantly associated with RFS (*p* < 0.05; Table 2). Among the clinicopathological factors, histopathological classification, Lauren classification, T stage, N stage, and adjuvant chemotherapy were significant predictors for RFS (*p* < 0.05). Variables that revealed statistical significance in the univariate analysis were incorporated into the multivariate survival analysis for RFS. In the multivariate survival analysis, only GLIM-defined malnutrition (*p* = 0.008; hazard ratio, 2.016; 95% CI, 1.198–3.395) and the ΔVAT index (*p* = 0.008; hazard ratio, 2.191; 95% CI, 1.230–3.904) were independent predictors for RFS, along with T stage and N stage (Table 2). The malnourished group (56.2%; 95% CI, 44.1–68.3%) and patients with a marked loss of VAT index (64.9%; 95% CI, 55.1–74.7%) showed significantly worse 5-year RFS rates than the non-malnourished group (81.5%; 95% CI, 76.2–86.8%) and those with a smaller loss of VAT index (81.2%; 95% CI, 75.5–86.9%), respectively (*p* < 0.001; Figure 3).

### 3.3. Stratification of RFS by Combining the GLIM Criteria and the ΔVAT Index

**Figure 4:**
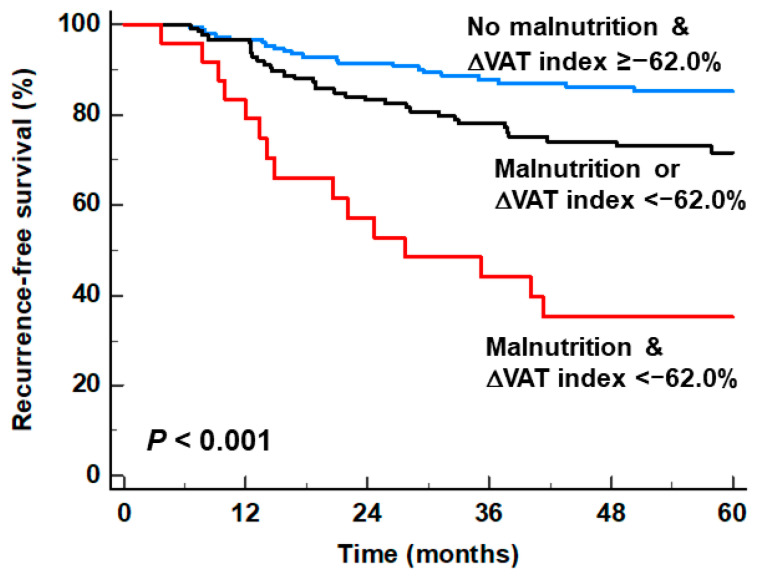
Cumulative recurrence-free survival curves stratified by combining the GLIM criteria and the ΔVAT index.

Combining the GLIM criteria and ΔVAT index further stratified the prognosis of the enrolled patients into three patient groups with significantly distinct RFS (patients with both malnutrition and a marked loss of VAT index, patients with either malnutrition or a marked loss of VAT index, and patients with no malnutrition and a smaller loss of VAT index). The malnutrition and ΔVAT index <−62.0% group (*p* < 0.001; hazard ratio, 6.791; 95% CI, 3.573–12.907) and the either malnutrition or ΔVAT index < −62.0% group (*p* = 0.006; hazard ratio, 2.082; 95% CI, 1.233–3.515) had significantly worse RFS than the no malnutrition and ΔVAT index ≥ −62.0% group (Table 3). Among the three patient groups, the malnutrition and marked loss of VAT index group showed the worst 5-year RFS rate of 35.2% (95% CI, 15.6–54.8%), followed by the either malnutrition or a marked loss of VAT index group (71.6%; 95% CI, 63.2–80.0%) and the no malnutrition and a smaller loss of VAT index group (85.3%; 95% CI, 79.4–91.8%; Figure 4).

## 4. Discussion

In the present study, we investigated whether changes in SAT and VAT areas during the first 6 months after surgery could further enhance the prognostic value of GLIM-defined malnutrition in AGC patients treated with curative radical gastrectomy. The GLIM criteria are comprised of essential nutritional information factors referenced from the established nutritional assessment tools and were originally developed to promote the global use of consensus malnutrition criteria in clinical practice [7,18]. Since the GLIM criteria were published in 2019, several studies have been performed to validate the clinical significance of the criteria in various kinds of cancers [7,18,19]. Because malnutrition is linked to diminished organ function and the systemic inflammatory status of the host, it is associated with treatment compliance, cancer progression, and survival in patients with malignant diseases [18,20,21]. In previous studies of gastric cancer patients, GLIM-defined malnutrition was found to be a poor prognostic factor for predicting disease-free survival and overall survival [6,10]. Similarly, our study also demonstrated that GLIM-defined malnutrition on preoperative assessment was an independent predictor for RFS in AGC patients treated with curative surgery. Recently, however, it has been pointed out that adipose tissue-related factors are not incorporated into the GLIM criteria [9,19]. Since adipose tissue plays a major role in reserving energy and the nutrition of the body, several studies have claimed that the addition of adipose tissue factors could significantly enhance the clinical value of the GLIM criteria [9,19,22].

Visceral adiposity has been shown to be an adverse factor for predicting post-operative complications and survival in several cancers including breast cancer, pancreatic cancer, and colorectal cancer [23,24,25,26]. For patients with gastric cancer, a high VAT index on preoperative CT images was also related to increased post-operative complications such as infection and leakage [27]; however, paradoxically, several previous studies demonstrated that obese patients had significantly better survival than non-obese patients and that a low VAT index was a poor prognostic factor for clinical outcomes in gastric cancer [9,11,27,28]. Similarly, in our study, a low VAT index on preoperative and 6-month follow-up CT scans was significantly associated with worse RFS in the univariate survival analysis, even though they failed to show statistical significance in the multivariate analysis. This paradoxical relationship, the so-called obesity paradox, is considered to result from the finding that the VAT mass reflects the amount of surplus energy and nutrition stored in the body [11,22,27]. Therefore, pretreatment low VAT mass could be another biomarker of pretreatment malnutrition, which is linked to poor prognosis [9,11,27]. Moreover, the role of VAT in controlling metabolic and immunologic activities has been suggested as an additional underlying mechanism between high VAT mass and favorable prognosis [9,28].

In the present study, 89.4% of the enrolled patients showed a decreased VAT index on their 6-month follow-up CT scans with a median ΔVAT index of −52.0%. In patients with gastric cancer, weight loss after radical surgery is regarded as a major problem [14,29]. In a nationwide cohort study conducted in Korea, 87.6% of patients with gastric cancer experienced post-operative weight loss [14]. Maximum weight loss was observed 1 year after surgery, and loss of VAT mass also manifested its maximum loss at 1 year after surgery and, thereafter, continuously slowly increased [29]. Weight loss can result in positive effects on the health of gastric cancer patients, for example, by decreasing the incidence of type 2 diabetes mellitus [14]. However, in previous studies, a marked loss of VAT mass after treatment was significantly associated with poor clinical outcomes of gastric cancer; this was also confirmed in our study [5,11]. There are two possible explanations for this association. First, since the amount of adipose tissue reflects the nutritional status of patients and a loss of adipose tissue occurs prior to that of skeletal muscle in the early phase of progressive metabolic disorder, a loss of adipose tissue mass has been suggested as a potential sensitive marker for cachectic body condition irrespective of skeletal muscle loss [30]. Therefore, a loss of VAT mass after surgery could reflect nutritional deficiency and a negative energy balance during the perioperative period [11,20]. This negative nutritional imbalance has been shown to be related to poor compliance with adjuvant chemotherapy and poor clinical outcomes in patients with gastric cancer [11,20]. Second, a chronic systemic inflammatory condition in patients could promote the loss of VAT [5,20]. A chronic systemic inflammatory condition could be a potential mechanism of the relationship between a loss of VAT and recurrence risk since the systemic inflammatory condition could contribute to a favorable host environment for cancer recurrence and is associated with an increased risk of cancer recurrence in patients with gastric cancer [20,31]. However, further studies are needed to explicitly elucidate the underlying mechanism between a loss of VAT after surgery and gastric cancer recurrence.

The results of our study demonstrated that the risk of cancer recurrence could be further stratified by combining GLIM-defined malnutrition and the degree of VAT mass loss. The patient subgroup who revealed malnutrition on preoperative assessment and a marked loss of the VAT index during the first 6 months after surgery showed the worst prognosis with a 5-year RFS rate of only 35.2%, whereas the patient subgroup with no malnutrition and a smaller loss of the VAT index had a 5-year RFS rate of 85.3%. These results suggest that not only preoperative nutritional status but also an alteration in nutritional status after surgery could be crucial factors for predicting the prognosis in patients with AGC. Hence, considering the prognostic significance of the loss of the VAT index, continuous nutritional surveillance with timely nutritional support is essential for AGC patients even after curative surgery [5]. Moreover, for AGC patients who were diagnosed with malnutrition in the preoperative assessment and showed markedly decreased VAT mass during follow-up, intensive treatment and surveillance strategies as well as active nutritional intervention would be needed due to the high risk of cancer recurrence. Since patients with <50 years showed a more prominent decrease in the SAT index and VAT index after the surgery, patients in the young age group might need more careful nutritional surveillance and support.

The present study has some limitations. First, because the study was a retrospective cohort study in a single medical center with a relatively small number of patients, there might be an inherent risk of selection bias. Further prospective studies with a larger patient population would be required to validate the results of our study. Second, because of the retrospective nature of the study, the interval between the surgery and the follow-up CT scan was not regular, which might affect our results. Finally, only an Asian population was included in this study. Since Asians are known to have a lower body index with a different adipose tissue distribution compared to Caucasians [27,32], the general application of our results to other racial groups might not be advisable.

## 5. Conclusions

GLIM-defined malnutrition diagnosed before surgery and a severe loss of VAT index during the first 6 months after surgery were independent predictors for RFS in AGC patients treated with curative surgery. The combination of GLIM-defined malnutrition and a loss of the VAT index further stratified the risk of gastric cancer recurrence, showing the worst RFS in patients with both malnutrition and a marked loss of the VAT index. In addition to initial nutritional assessment before surgery, post-operative surveillance of VAT mass change could provide information regarding the necessity of nutritional support and clinical outcomes.

## Figures and Tables

**Table 1:** Patient characteristics.

Characteristics	Total (*n* = 302)	Patients with Events (*n* = 75)	Patients with No Events (*n* = 227)	*p*-Value
Age (years)	60 (31–91)	60 (31–80)	60 (32–91)	0.409
Sex				0.437
Men	219 (72.5%)	57 (76.0%)	162 (71.4%)	
Women	83 (27.5%)	18 (24.0%)	65 (28.6%)	
Body mass index (kg/m^2^)	23.6 (16.0–34.6)	23.8 (16.2–33.3)	23.6 (16.0–34.6)	0.842
Obesity				0.365
Underweight	35 (11.6%)	12 (16.0%)	23 (10.1%)	
Normal	187 (61.9%)	43 (57.3%)	144 (63.4%)	
Obese	80 (26.5%)	20 (26.7%)	60 (26.4%)	
Tumor location				0.227
Upper	68 (22.5%)	22 (29.3%)	46 (20.3%)	
Middle	34 (11.3%)	9 (12.0%)	25 (11.0%)	
Lower	200 (66.2%)	44 (58.7%)	156 (68.7%)	
Histopathological classification				0.114
Papillary/tubular type	107 (35.4%)	18 (24.0%)	89 (39.2%)	
Poorly cohesive type	172 (57.0%)	51 (68.0%)	121 (53.3%)	
Mucinous	14 (4.6%)	4 (5.3%)	10 (4.4%)	
Others	9 (3.0%)	2 (2.7%)	7 (3.1%)	
Lauren classification				0.002 *
Intestinal type	104 (34.4%)	15 (20.0%)	89 (39.2%)	
Diffuse/indeterminate type	198 (65.6%)	60 (80.0%)	138 (60.8%)	
Surgery type				0.006 *
Total gastrectomy	80 (26.5%)	30 (40.0%)	50 (22.0%)	
Subtotal gastrectomy	218 (72.2%)	45 (60.0%)	173 (76.2%)	
Proximal gastrectomy	4 (1.3%)	0 (0.0%)	4 (1.8%)	
T stage				<0.001 *
T2 stage	94 (31.1%)	4 (5.3%)	90 (39.6%)	
T3 stage	135 (44.7%)	36 (48.0%)	99 (43.6%)	
T4 stage	73 (24.2%)	35 (46.7%)	38 (16.7%)	
N stage				<0.001 *
N0 stage	127 (42.1%)	9 (12.0%)	118 (52.0%)	
N1–3 stage	175 (57.9%)	66 (88.0%)	109 (48.0%)	
TNM stage				<0.001 *
Stage I	59 (19.5%)	1 (1.3%)	58 (25.6%)	
Stage II	126 (41.7%)	16 (21.3%)	110 (48.5%)	
Stage III	117 (38.7%)	58 (77.3%)	59 (26.0%)	
Adjuvant chemotherapy				<0.001 *
Yes	263 (87.1%)	74 (98.7%)	189 (83.3%)	
No	39 (12.9%)	1 (1.3%)	38 (16.7%)	
GLIM criteria				<0.001 *
Malnutrition	70 (23.2%)	31 (41.3%)	39 (17.2%)	
No malnutrition	232 (76.8%)	44 (58.7%)	188 (82.8%)	
Initial SAT index (cm^2^/m^2^)	45.5 (2.2–162.3)	42.1 (2.2–142.6)	45.8 (4.4–162.3)	0.649
Initial VAT index (cm^2^/m^2^)	43.5 (0.9–147.8)	38.0 (2.8–147.8)	44.6 (0.9–140.9)	0.217
Follow-up SAT index (cm^2^/m^2^)	30.7 (1.0–111.6)	26.1 (1.6–111.6)	32.4 (1.0–101.2)	0.007 *
Follow-up VAT index (cm^2^/m^2^)	19.5 (1.2–103.1)	12.8 (1.2–103.1)	21.4 (1.2–72.3)	0.012 *
ΔSAT index (%)	−26.3 (−97.4–235.5)	−35.5 (−86.2–220.5)	−23.9 (−97.4–235.5)	0.005 *
ΔVAT index (%)	−52.0 (−98.6–369.7)	−59.7 (−93.5–139.7)	−48.8 (−98.6–369.7)	0.006 *

Data are expressed as patient number (%) or median (range). * *p*-value < 0.05. GLIM, Global Leadership Initiative on Malnutrition; SAT, subcutaneous adipose tissue; VAT, visceral adipose tissue; Events, recurrence or death from cancer.

**Table 2:** Univariate and multivariate Cox regression analyses for recurrence-free survival.

Variables		Univariate Analysis		Multivariate Analysis	
		*p*-Value	Hazard Ratio (95% CI)	*p*-Value	Hazard Ratio (95% CI)
Age (<50 years vs.)	50–70 years	0.795	0.924 (0.514–1.665)		
	>70 years	0.876	1.057 (0.527–2.119)		
Sex (women vs. men)		0.432	1.237 (0.728–2.102)		
Obesity (underweight vs.)	Normal	0.151	0.626 (0.330–1.187)		
	Obese	0.284	0.676 (0.330–1.383)		
Tumor location (upper vs.)	Middle	0.648	0.835 (0.384–1.813)		
	Lower	0.106	0.655 (0.392–1.093)		
Histopathological classification (papillary/tubular type vs.)	Poorly cohesive type	0.027 *	1.839 (1.074–3.149)	0.727	0.879 (0.425–1.818)
	Mucinous	0.339	1.698 (0.574–5.020)	0.812	0.871 (0.280–2.712)
	Others	0.453	1.750 (0.406–7.548)	0.664	0.717 (0.160–3.208)
Lauren classification (Intestinal vs. diffuse/indeterminate)		0.004 *	2.320 (1.317–4.087)	0.153	1.764 (0.810–3.838)
T stage (T2 vs.)	T3	<0.001 *	6.850 (2.437–19.251)	0.004 *	4.859 (1.680–14.052)
	T4	<0.001 *	16.942 (6.009–47.767)	<0.001 *	9.917 (3.355–29.316)
N stage (N0 vs. N1–3)		<0.001 *	6.449 (3.212–12.946)	<0.001 *	4.234 (2.071–8.654)
Adjuvant chemotherapy (no vs. yes)		0.016 *	11.363 (1.579–81.774)	0.577	1.777 (0.236–13.397)
GLIM criteria (no malnutrition vs. malnutrition)		<0.001 *	2.695 (1.701–4.271)	0.008 *	2.016 (1.198–3.395)
Initial SAT index (≥43.6 vs. <43.6)		0.113	1.445 (0.917–2.277)		
Initial VAT index (≥39.5 vs. <39.5)		0.012 *	1.797 (1.139–2.836)	0.753	1.102 (0.602–2.019)
Follow-up SAT index (≥29.5 vs. <29.5)		0.007 *	1.888 (1.190–2.998)	0.997	0.999 (0.538–1.857)
Follow-up VAT index (≥11.1 vs. <11.1)		<0.001 *	2.561 (1.625–4.036)	0.534	0.804 (0.403–1.602)
ΔSAT index (≥−41.6 vs. <−41.6)		0.002 *	2.082 (1.317–3.292)	0.146	1.569 (0.855–2.881)
ΔVAT index (≥−62.0 vs. <−62.0)		<0.001 *	2.168 (1.378–3.411)	0.008 *	2.191 (1.230–3.904)

* *p*-value < 0.05. CI, confidence interval; GLIM, Global Leadership Initiative on Malnutrition; SAT, subcutaneous adipose tissue; VAT, visceral adipose tissue.

**Table 3:** Comparisons of recurrence-free survival according to the combination of the GLIM criteria and the ΔVAT index.

Patient Subgroup	Number of Events (%)	*p*-Value	Hazard Ratio (95% CI)
No malnutrition & ΔVAT index ≥ −62.0% (*n* = 152)	23 (15.1%)	-	1.00
Malnutrition or ΔVAT index < −62.0% (*n* = 126)	36 (28.6%)	0.006	2.082 (1.233–3.515)
Malnutrition & ΔVAT index < −62.0% (*n* = 24)	16 (66.7%)	<0.001	6.791 (3.573–12.907)

CI, confidence interval; GLIM, Global Leadership Initiative on Malnutrition; VAT, visceral adipose tissue; Events, recurrence or death from cancer.

## Data Availability

The datasets generated during and/or analyzed during the current study are available from the corresponding authors upon reasonable request.

## Supplementary Materials

The following supporting information can be downloaded at: https://www.mdpi.com/article/10.3390/nu16050728/s1, Table S1: Comparison of adipose tissue index parameters according to age groups of enrolled patients.
