# Supplementary material for: Adipose Tissue Quantification Improves the Prognostic Value of GLIM Criteria in Advanced Gastric Cancer Patients
Source: Nutrients. 2024 Mar 2;16(5):728. doi: 10.3390/nu16050728 (PMC10934376; doi:10.3390/nu16050728)
Supplement: Supplementary file 1 [file nutrients-16-00728-s001.zip › nutrients-2823475-supplementary.pdf]

Table S1. Comparisons of adipose tissue index parameters according to age groups of enrolled patients.

Adipose tissue index parameters	Age groups			P-value
	<50 years	50–70 years	>70 years	
Initial SAT index (cm^2/m^2)	52.1 (32.9–71.0)	43.6 (30.3–54.3)	45.7 (27.6–64.2)	0.025*
Initial VAT index (cm^2/m^2)	40.7 (27.8–56.5)	43.6 (26.7–62.7)	48.7 (31.0–76.7)	0.255
Follow-up SAT index (cm^2/m^2)	30.3 (21.9–46.1)	30.5 (20.1–42.6)	34.4 (22.5–50.1)	0.348
Follow-up VAT index (cm^2/m^2)	13.0 (6.3–23.5)	20.8 (10.8–34.3)	26.9 (12.7–36.3)	0.002†
ΔSAT index (%)	-36.2 (-54.8– -11.2)	-29.1 (-44.8–0.1)	-17.7 (-40.3–0.7)	0.015‡
ΔVAT index (%)	-57.6 (-75.2– -40.9)	-51.8 (-67.7– -30.1)	-48.7 (-61.7– -25.0)	0.016‡

All values are expressed in median (interquartile range)

SAT, subcutaneous adipose tissue; VAT, visceral adipose tissue

*On post-hoc analysis, patients with <50 years had significant higher values than those with 50–70 years (p<0.05)

†On post-hoc analysis, patients with 50–70 years and >70 years had significant higher values than those with <50 years (p<0.05)

‡On post-hoc analysis, patients with >70 years had significantly higher values than those with <50 years (p<0.05)
